# Prognostic Value of Different CMR-Based Techniques to Assess Left Ventricular Myocardial Strain in Takotsubo Syndrome

**DOI:** 10.3390/jcm9123882

**Published:** 2020-11-29

**Authors:** Thomas Stiermaier, Kira Busch, Torben Lange, Toni Pätz, Moritz Meusel, Sören J. Backhaus, Alex Frydrychowicz, Jörg Barkhausen, Matthias Gutberlet, Holger Thiele, Andreas Schuster, Ingo Eitel

**Affiliations:** 1 Medical Clinic II, University Heart Center Luebeck, 23538 Luebeck, Germany; kirabusch@gmx.de (K.B.); toni.paetz@uksh.de (T.P.); moritz.meusel@uksh.de (M.M.); ingo.eitel@uksh.de (I.E.); 2 German Center for Cardiovascular Research (DZHK), Partner Site Hamburg/Kiel/Luebeck, 23538 Luebeck, Germany; 3 Department of Cardiology and Pneumology, Georg-August University, University Medical Center Goettingen, 37075 Goettingen, Germany; torben.lange@med.uni-goettingen.de (T.L.); soeren.backhaus@med.uni-goettingen.de (S.J.B.); andreas.schuster@med.uni-goettingen.de (A.S.); 4 German Center for Cardiovascular Research (DZHK), Partner Site Goettingen, 37075 Goettingen, Germany; 5 Department of Radiology and Nuclear Medicine, University Hospital Schleswig-Holstein, Campus Luebeck, 23538 Luebeck, Germany; alex.frydrychowicz@uksh.de (A.F.); joerg.barkhausen@uksh.de (J.B.); 6 Heart Center Leipzig at University of Leipzig, Department of Radiology, 04289 Leipzig, Germany; matthias.gutberlet@medizin.uni-leipzig.de; 7 Department of Internal Medicine/Cardiology and Leipzig Heart Institute, Heart Center Leipzig at University of Leipzig, 04289 Leipzig, Germany; holger.thiele@medizin.uni-leipzig.de

**Keywords:** takotsubo syndrome, cardiac magnetic resonance, myocardial strain, prognosis

## Abstract

Cardiac magnetic resonance (CMR)-derived left ventricular (LV) global longitudinal strain (GLS) provides incremental prognostic information on various cardiovascular diseases but has not yet been investigated comprehensively in patients with Takotsubo syndrome (TS). This study evaluated the prognostic value of feature tracking (FT) GLS, tissue tracking (TT) GLS, and fast manual long axis strain (LAS) in 147 patients with TS, who underwent CMR at a median of 2 days after admission. Long-term mortality was assessed 3 years after the acute event. In contrast to LV ejection fraction and tissue characteristics, impaired FT-GLS, TT-GLS and fast manual LAS were associated with adverse outcome. The best cutoff points for the prediction of long-term mortality were similar with all three approaches: FT-GLS −11.28%, TT-GLS −11.45%, and fast manual LAS −10.86%. Long-term mortality rates were significantly higher in patients with FT-GLS > −11.28% (25.0% versus 9.8%; *p* = 0.029), TT-GLS > −11.45% (20.0% versus 5.4%; *p* = 0.016), and LAS > −10.86% (23.3% versus 6.6%; *p* = 0.014). However, in multivariable analysis, diabetes mellitus (*p* = 0.001), atrial fibrillation (*p* = 0.001), malignancy (*p* = 0.006), and physical triggers (*p* = 0.006) outperformed measures of myocardial strain and emerged as the strongest, independent predictors of long-term mortality in TS. In conclusion, CMR-based longitudinal strain provides valuable prognostic information in patients with TS, regardless of the utilized technique of assessment. Long-term mortality, however, is mainly determined by comorbidities.

## 1. Introduction

Takotsubo syndrome (TS) is an increasingly recognized form of acute heart failure and important differential diagnosis in patients presenting with suspected acute coronary syndrome. A characteristic and unique feature of TS is the distinctive contraction pattern of the left ventricle during the acute phase of the disease [1]. The regional distribution of circumferential akinesia and hypercontraction results in apical, midventricular, or basal ballooning, which is rapidly reversible within several days to weeks. Therefore, the visualization and quantification of left ventricular (LV) dysfunction plays a key role in the diagnosis and management of patients with TS. In clinical routine, measurement of LV ejection fraction is the most popular method to assess ventricular performance [2]. In TS, however, ejection fraction is often only moderately reduced since regional hypercontraction balances the pronounced wall motion abnormalities. Consequently, ejection fraction does not adequately reflect the extent of LV systolic dysfunction in TS and has limited prognostic value for the prediction of adverse clinical outcome [3,4]. Alternative approaches for a more accurate determination of ventricular function in TS are needed. Cardiac magnetic resonance (CMR) provides comprehensive insights into structural and functional myocardial pathologies and is uniquely suited to reliably diagnose TS at an early stage of the disease by excluding potential differential diagnoses [5]. Recently, CMR-based techniques for the multidirectional assessment of myocardial deformation proved to be superior measures of LV performance and, in particular, the LV longitudinal strain emerged as a strong prognostic marker in various cardiovascular diseases [6,7,8,9,10,11]. Based on the findings of a small study, in which persistently impaired LV longitudinal strain was reported despite normalization of ejection fraction in 36 patients with TS [12], it can be hypothesized that deformation indices are superior prognostic markers in TS. However, different techniques and software solutions to measure longitudinal strain have been proposed and their actual value for risk stratification in patients with TS is unknown [6,13,14,15].

The aim of this study was, therefore, to investigate different approaches for the assessment of longitudinal strain using standard steady-state free precession (SSFP) CMR images and determine their value for the prediction of adverse outcome following TS.

## 2. Methods

### 2.1. Study Population

The population of this cohort study consisted of 147 patients with TS prospectively enrolled at the Heart Center Leipzig (*n* = 125) and the University Heart Center Luebeck (*n* = 22). All patients underwent CMR imaging during the acute phase of the disease and met the recommended clinical and CMR criteria for the diagnosis of TS including the absence of a culprit lesion in coronary angiography and complete recovery of LV systolic function [1,5,16,17]. Triggering factors for TS were divided into emotional/psychic stress and physical stressors due to adjuvant organic conditions (e.g., surgery, severe trauma, bronchospasm, sepsis, etc.). Clinical outcome data were acquired during regular outpatient visits or via telephone contact with the patients, relatives, and treating physicians. The study endpoint in the present analysis was all-cause mortality. All events were verified via medical records and finally adjudicated by a clinical events committee.

The study was conducted according to the principles of the Declaration of Helsinki and approved by the local ethical committees. All patients gave written informed consent.

### 2.2. CMR Image Acquisition and Analysis

CMR scans were performed on clinical 1.5- or 3.0-T magnetic resonance scanners. The standardized protocol included ECG-gated balanced SSFP sequences of 2- and 4-chamber long-axis views as well as short axis stacks for functional analysis. The presence of myocardial edema was assessed in T2-weighted triple short-tau inversion recovery images and T1-weighted inversion-recovery-gradient echo sequences were acquired 10 to 15 min after administration of a gadolinium bolus (late gadolinium enhancement imaging with individually adjusted inversion times) to determine the presence of myocardial scar/fibrosis. LV ejection fraction, presence of myocardial edema, and myocardial fibrosis were assessed in short axis stacks from base to apex. Analyses were performed offline by blinded investigators at the CMR core-laboratory at University Heart Center Luebeck using certified evaluation software (cmr42, Circle Cardiovascular Imaging Inc., Calgary, AB, Canada). T2 signal intensity ratios were calculated by comparing the mean signal intensity of the myocardium with that of the skeletal muscle in the same slice. A T2 signal intensity ratio ≥1.9 was the predefined threshold to identify myocardial edema. Significant late gadolinium enhancement was defined as >5 standard deviations above normal myocardium.

### 2.3. CMR-Based Strain Analysis

**Figure 1:**
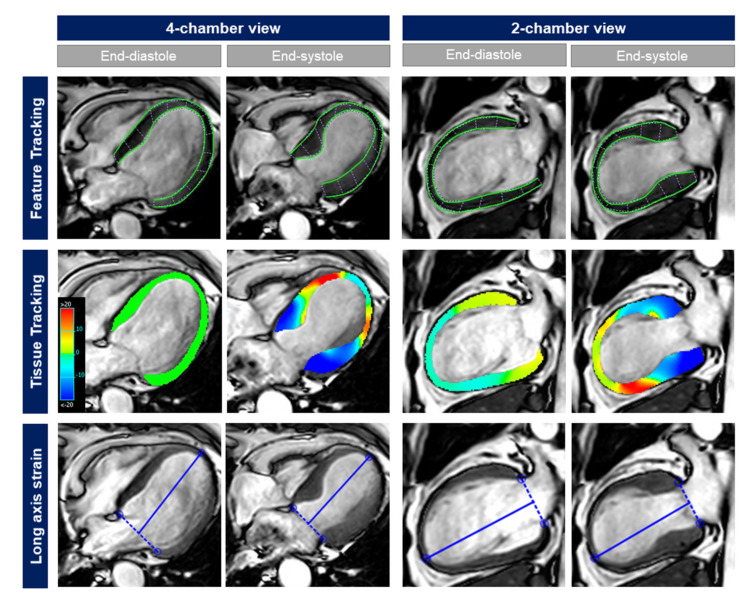
Different CMR-based approaches for the assessment of LV longitudinal strain in a 68-year old female patient presenting with TS and typical apical ballooning. While LV ejection fraction was only moderately reduced (42%), FT-GLS (−7.9%), TT-GLS (−8.1%), and fast manual LAS (−9.6%) showed a severely impaired longitudinal strain.

Central core-laboratory masked analyses included 3 different approaches for the assessment of LV longitudinal strain: (a) global longitudinal strain (GLS) assessed with feature tracking (FT-GLS); (b) GLS assessed with tissue tracking (TT-GLS); and (c) fast manual long-axis strain (LAS). Examples are provided in Figure 1.

CMR feature tracking was performed in an experienced core-laboratory at the University Medical Center Goettingen using dedicated evaluation software that has been validated and previously used in numerous studies (2D CPA MR, Cardiac Performance Analysis, Version 1.1.2, TomTec Imaging Systems, Unterschleissheim, Germany) [6,13,18]. LV endocardial borders were manually traced at end-diastole in 2- and 4-chamber SSFP long-axis sequences using a point-and-click approach. Subsequently, an automatic border tracking algorithm was applied, which tracks image features throughout the cardiac cycle. Visual review and manual adjustments assured accurate tracking with consequent reapplication of the algorithm if necessary. Average strain values from basal, midventricular, and apical segments were used to determine FT-GLS based on the average of 3 repeated, independent analyses. TT-GLS and fast manual LAS were determined in the core-laboratory at the University Heart Center Luebeck using cmr42 (Circle Cardiovascular Imaging Inc., Calgary, AB, Canada). For the assessment of TT-GLS, end-diastolic endocardial and epicardial LV contours were manually drawn in 2- and 4-chamber SSFP long-axis images, enabling the software to semi-automatically track the myocardium throughout the cardiac cycle [14]. Fast manual LAS was calculated as described previously based on the average of 2- and 4-chamber SSFP sequences [13,19]. In brief, the distance between the epicardial apical border and the middle of a line connecting the origins of the mitral leaflets was measured in end-diastole and end-systole. The difference was divided by the end-diastolic length and multiplied by 100 to calculate percent strain values.

All analyses were performed by experienced and fully blinded operators at core laboratories with low inter- and intra-observer variabilities, as previously demonstrated [2,13].

### 2.4. Statistical Analysis

Categorical variables are presented as frequencies (percentages) and were compared with the Chi-square test. Continuous variables were mainly non-normally distributed in Shapiro–Wilk test and are therefore presented as medians with corresponding interquartile ranges (IQR). Between group differences were assessed with the Mann–Whitney U test. Baseline characteristics and CMR findings are reported for the entire study population and were compared between survivors and non-survivors. Correlations were assessed between LV ejection fraction and the different approaches to determine myocardial strain using the Spearman’s rank correlation coefficient. Intra- and inter-observer reproducibility in the core laboratories was tested in 10 randomly selected patients with TS. Calculations included Bland–Altman analysis, intraclass correlation coefficients (ICC), and coefficients of variation (CoV) using the root mean square method. The level of agreement was considered excellent in case of an ICC > 0.74. After application of receiver-operating characteristic curves, the Youden index was calculated to dichotomize myocardial strain and determine the best cutoff values for the prediction of outcome. These thresholds were used to stratify the study population and assess the impact of myocardial strain values on long-term mortality with the Kaplan–Meier method and log-rank testing. Predictors of mortality were identified in univariate and stepwise multivariable Cox regression analysis. Hazard ratios (HR) with corresponding 95% confidence intervals (CI) are reported. All baseline characteristics and CMR findings were considered for univariate analysis. Multivariable testing included only significant predictors of mortality in univariate analysis (*p* < 0.05). Furthermore, strain values and their prognostic implications were also assessed regarding confirmed or presumed cardiovascular causes of death.

Statistical analyses were performed with SPSS version 23.0 (IBM, Armonk, New York, NY, USA) and MedCalc version 19.2.1. (MedCalc Software, Ostend, Belgium). A 2-tailed *p*-value < 0.05 was considered statistically significant.

## 3. Results

The study population consisted of 147 consecutive patients with TS who underwent CMR imaging at a median of 2 days (IQR 2 to 4 days) after hospitalization. Clinical follow-up data after 3 years (IQR 1.6 to 5.3 years) were available in 138 patients (94%) and showed an all-cause mortality of 16% (cardiovascular causes, 26.3%; non-cardiovascular causes, 36.8%; unknown cause of death, 36.8%).

### 3.1. Baseline Characteristics and Routine CMR Findings

The investigated cohort reflected a typical TS population of predominantly postmenopausal women with a stressful trigger in two-thirds of patients (Table 1).

Apical ballooning was the most prevalent contraction pattern resulting in a moderately reduced LV ejection fraction of 47% (IQR 41 to 53%) at acute presentation. CMR imaging performed a few days after admission still demonstrated a comparable impairment of LV performance (Table 2).

Furthermore, CMR revealed myocardial edema in 80% of patients and excluded significant scar/fibrosis. Complete recovery of LV function was documented in all participants.

Non-survivors during follow-up were significantly older (*p* = 0.006), more frequently male (*p* = 0.040), had more physical triggers (*p* = 0.002) and a higher prevalence of diabetes mellitus (*p* = 0.011) and malignancies (*p* = 0.003; Table 1). Moreover, the number of patients with atrial fibrillation (*p* = 0.023) and consequently oral anticoagulation (*p* = 0.034) was significantly higher among non-survivors. Discharge medication differed also regarding the use of diuretics, which was higher in deceased TS patients (*p* = 0.030). Routine CMR findings including LV ejection were similar among survivors and non-survivors (Table 2).

### 3.2. CMR-Bases Strain Analysis

**Figure 2:**
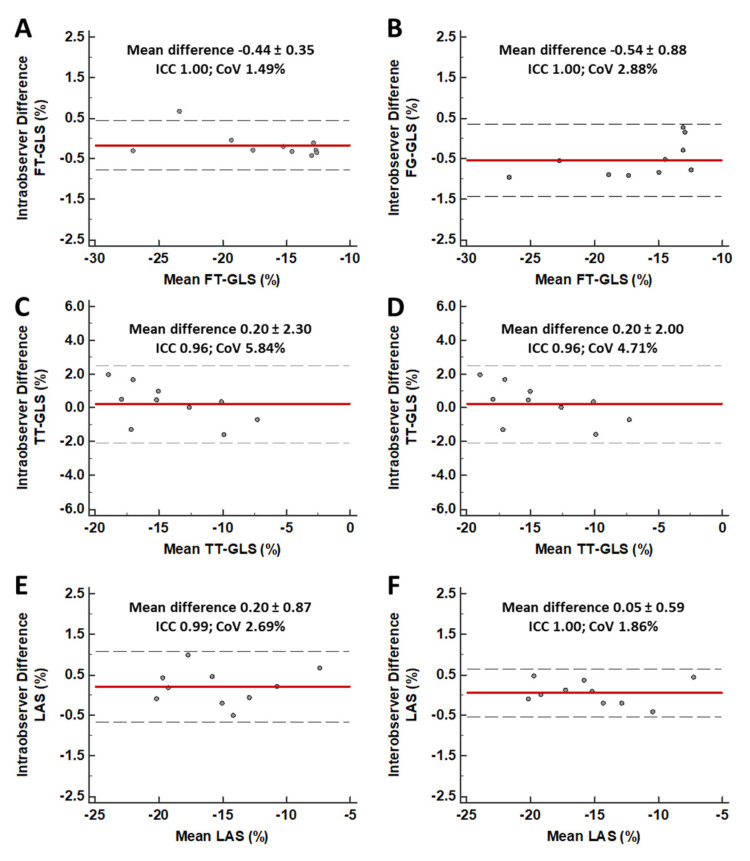
Intra- and Inter-observer variability of different approaches for strain assessment. Bland–Altman analysis showing low intra- and inter-observer variability for FT-GLS (Panels **A** and **B**), TT-GLS (Panels **C** and **D**), and fast manual LAS (Panes **E** and **F**). Red line = mean absolute difference; Black, dotted lines = 95% confidence interval. CoV = Coefficient of variation; FT = feature tracking; GLS = global longitudinal strain; ICC = Intraclass correlation coefficient; LAS = long-axis strain; TT = tissue tracking.

The different CMR-based approaches to assess LV myocardial deformation resulted in the following strain values: FT-GLS −14.2% (IQR −11.3 to −18.2%), TT-GLS −10.4% (−8.6% to −12.8%), and fast manual LAS −11.2% (−9.1 to −14.2%). All strain parameters demonstrated excellent intra- and inter-observer reproducibility (Figure 2) and a moderate but significant correlation with LV ejection fraction (FT-GLS: *r* = −0.525; TT-GLS: *r* = −0.674; LAS: *r* = −0.514; *p* < 0.001 for all).

**Figure 3:**
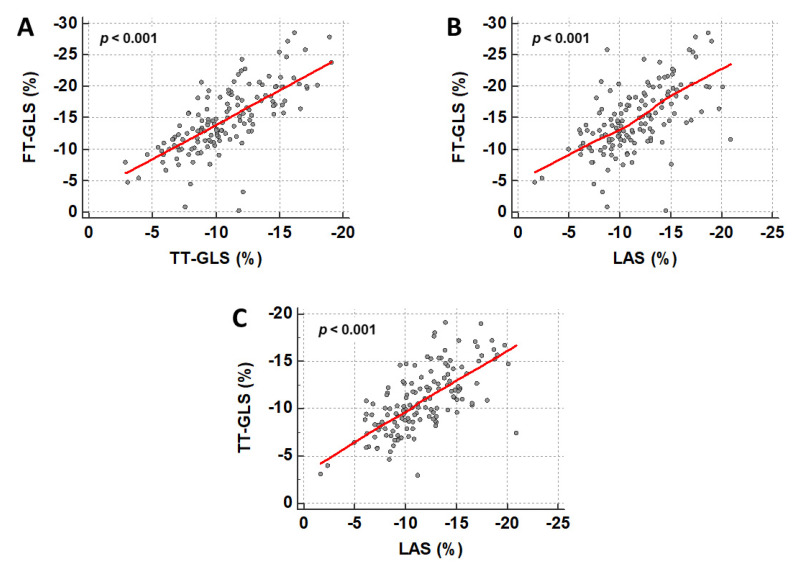
Correlation between different CMR-based approaches for strain assessment. Scatter plots show a significant correlation between FT-GLS and TT-GLS (**A**), FT-GLS and LAS (**B**), and TT-GLS and LAS (**C**). FT = feature tracking; GLS = global longitudinal strain; LAS = long-axis strain; TT = tissue tracking.

The relationship between the different deformation indices is illustrated in Figure 3 and showed a stronger correlation (FT-GLS and TT-GLS: *r* = 0.750; FT-GLS and LAS: *r* = 0.602; TT-GLS and LAS: *r* = 0.675; *p* < 0.001 for all). TT-GLS (*p* = 0.032) and fast manual LAS (*p* = 0.014) were significantly impaired in non-survivors, and FT-GLS showed a trend towards higher values in survivors (*p* = 0.097; Table 2).

### 3.3. Prognostic Value of CMR-Based Strain Values

**Figure 4:**
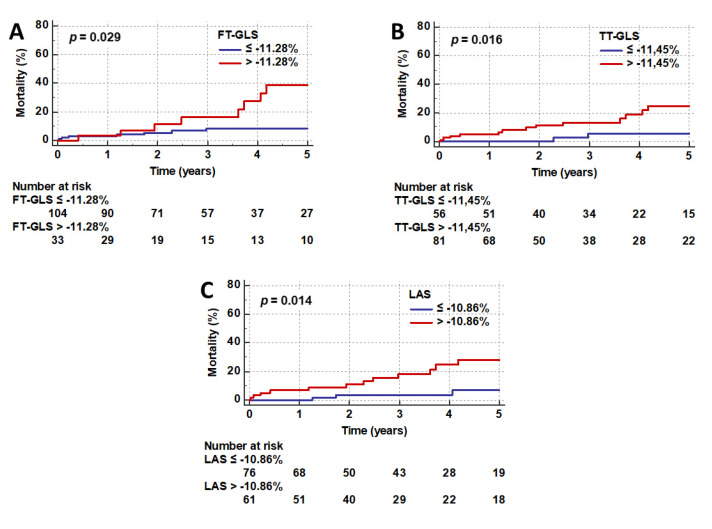
Kaplan–Meier plots. Long-term mortality rates after stratification according to the best cutoff values for FT-GLS (**A**), TT-GLS (**B**) and fast manual LAS (**C**). *p*-values were calculated with log-rank testing. FT = feature tracking; GLS = global longitudinal strain; LAS = long-axis strain; TT = tissue tracking.

The following values were identified as the best cutoff points for the prediction of outcome in TS: FT-GLS −11.28%, TT-GLS −11.45%, and fast manual LAS −10.86%. The results of Kaplan–Meier analysis and log-rank testing after dichotomizing the study population according to these thresholds are reported in Figure 4.

The observed long-term mortality rates were significantly higher in patients with FT-GLS > −11.28% (25.0% versus 9.8%; *p* = 0.029; Figure 4A), TT-GLS > −11.45% (20.0% versus 5.4%; *p* = 0.016; Figure 4B), and LAS > −10.86% (23.3% versus 6.6%; *p* = 0.014; Figure 4C).

Univariate Cox regression analysis confirmed the association with mortality for all three approaches (Table 3), while LV ejection fraction did not predict outcome (*p* = 0.372). However, when also including clinical markers in a multivariable model, myocardial strain was no longer independently associated with adverse outcome and diabetes mellitus (*p* = 0.001), atrial fibrillation (*p* = 0.001), malignancy (*p* = 0.006), and physical triggers (*p* = 0.006) emerged as the strongest, independent predictors of mortality in patients with TS (Table 3).

An additional analysis, which excluded non-cardiovascular causes of death, revealed impaired FT-GLS (−11.2% (IQR −9.1% to –15.6%) versus −14.5% (IQR −11.5% to −18.5%); *p* = 0.052), TT-GLS (−8.6% (IQR −6.7% to −10.0%) versus −10.9% (IQR −8.9% to −13.2%); *p* = 0.013), and LAS (−9.5% (IQR −6.5% to −10.8%) versus −12.0% (IQR −9.4% to −14.5%); *p* = 0.006) in patients with documented or presumed cardiovascular death. Univariate regression analysis confirmed the association of all strain parameters with cardiovascular outcome (FT-GLS: HR 1.13 (95% CI 1.02 to 1.27) *p* = 0.026; TT-GLS: HR 1.26 (95% CI 1.03 to 1.53), *p* = 0.023; LAS: HR 1.29 (95% CI 1.08 to 1.54), *p* = 0.005) and TT-GLS remained an independent predictor of cardiovascular death (HR 1.26, 95% CI 1.00 to 1.59; *p* = 0.052) in addition to age (*p* = 0.055) and atrial fibrillation (*p* = 0.032) in multivariable testing.

## 4. Discussion

The present study is the first to comprehensively investigate different CMR-based approaches for the assessment of LV longitudinal strain in patients with acute TS regarding their value for long-term risk stratification. The main results can be summarized as follows: (a) in patients with TS, LV longitudinal strain is a superior marker of clinical outcome compared to LV ejection fraction; (b) FT-GLS, TT-GLS, and fast manual LAS provide similar prognostic information with a cutoff at approximately −11% to identify high-risk patients; and (c) long-term mortality in TS is mainly determined by comorbidities, which outperformed measures of myocardial strain in multivariable analysis.

Despite the transient character of TS, patients face a substantial risk of life-threatening complications during the acute phase of the disease and the reported long-term mortality rates are at least comparable to patients with acute myocardial infarction [3,20,21]. Therefore, clinical research in TS focuses increasingly on prognostic markers to identify high-risk patients with adverse outcome. Multimodality imaging plays a key role for establishing the diagnosis and guiding the therapy of patients with TS [17]. However, the value of imaging parameters for the prediction of outcome is still under investigation and, in particular, LV ejection fraction, an established prognostic marker in several cardiovascular diseases, showed inconsistent results in TS populations. While a lower LV ejection fraction was clearly associated with the occurrence of cardiogenic shock [22,23], long-term prognostic implications were not consistently evident in clinical studies [4]. Potential explanations for these negative results are that systolic LV function is only temporarily impaired in TS and/or that global ejection fraction does not adequately reflect the focal contraction abnormalities in TS and overestimates LV performance because of hypercontractility in non-affected regions of the LV. These drawbacks of LV ejection fraction might be overcome by the assessment of multidirectional myocardial deformation indices. In particular, CMR-based approaches to determine LV longitudinal strain emerged as superior functional markers for risk stratification in various diseases across the cardiovascular continuum [6,7,8]. In the present study, we investigated three different techniques to analyze longitudinal strain in routinely acquired balanced SSFP images without the need for additional sequences. CMR-FT (endocardial-cavity border) and CMR-TT (software-generated myocardial notes) follow the motion of myocardial characteristics to track myocardial deformation and require dedicated software solutions [24,25,26]. In contrast, LAS enables a fast, easy, and software-independent approximation of longitudinal shortening without the need for costly licensing and time-consuming post-processing [13,19]. All three techniques demonstrated excellent inter- and intra-observer reproducibility in previous analyses and in our study investigating patients with TS [13,14,27]. Furthermore, our results show comparable longitudinal strain values and a strong correlation between the different approaches. Unlike LV ejection fraction, longitudinal strain derived from each technique was associated with long-term outcome, and, in addition, the calculated cut-off values for the identification of high-risk patients were similar with an ideal threshold of approximately −11%. Of note, the additional effort with a simple, widely available approach like fast manual LAS is low and CMR imaging is already recommended in patients with acute TS to rule out other common pathologies/differential diagnoses [17]. However, despite the association with outcome, our data do not show independent prognostic implications of LV strain beyond established clinical risk factors. Diabetes mellitus, atrial fibrillation, malignancies, and physical triggers emerged as the most powerful predictors of long-term mortality in TS and have also been identified as useful markers for risk stratification in previous studies [28,29,30,31,32]. These findings are not entirely surprising in view of a substantial contribution of non-cardiac causes of death in patients with TS [3]. LV longitudinal strain might hold a greater potential to improve risk assessment regarding cardiac endpoints. Patients with TS face a substantial risk of cardiovascular complications during the acute phase of the disease (e.g., severe heart failure resulting in pulmonary edema or cardiogenic shock; life-threatening arrhythmias, or LV thrombus formation) [20,22,33]. Since these events usually occur very early, CMR imaging has to be performed immediately after diagnosing TS, which might be limited by the available resources in some institutions. Another potential application of myocardial strain in TS is the identification of patients with residual contraction abnormalities, who are at risk for late arrhythmic events, ongoing heart failure symptoms or TS recurrence [34]. Previous CMR data show persistently impaired cardiac deformation indices in patients with prior TS despite normalization of LV ejection fraction and biomarkers [12]. This aspect could explain the association of GLS with outcome, while LV ejection fraction did not show a prognostic value. Therefore, CMR-based assessment of longitudinal strain might help to identify high-risk patients and improve their management (e.g., with intensified medical heart failure therapy) and consequently the outcome. However, currently available data including the findings of our trial can only be interpreted as hypothesis generating. Novel advances in deformation imaging techniques such as artificial intelligence-based fully automated FT or fast strain-encoded imaging (SENC) may additionally increase the diagnostic value of myocardial strain in TS and further facilitate the process [35,36]. Notwithstanding, prospective studies investigating treatment approaches according to deformation indices are necessary to prove a substantial benefit. In addition, more precise tissue characterization with mapping techniques might also provide prognostic information in TS and requires evaluation in future studies.

Limitations of the current study refer to the rather small and low-risk study population. CMR could not be performed in patients with contraindications (e.g., metallic implants or claustrophobia) and unstable patients. The time from diagnosing TS to CMR was, at median, 2 days and hence after the critical period of the first 24 to 48 h. Therefore, there is an unavoidable selection bias for stable patients without severe complications during the acute phase of the disease. The primary study endpoint was all-cause mortality with a considerable number of patients with unknown causes of death. The impact of longitudinal strain on early adverse events or cardiovascular mortality could not be assessed in detail. Furthermore, the effect of confounding factors (e.g., rhythm disorders) on myocardial strain was not assessed in our study. However, our study reports CMR data from one of the largest cohorts of patients with TS with long-term outcome data and we were able to show a prognostic value even in this low-risk population. Follow-up CMR was not performed routinely in our study. Data regarding persistently impaired cardiac deformation indices after TS are available from previous investigations [12].

## 5. Conclusions

This CMR study shows that different approaches to assess LV longitudinal strain, including a simple technique like fast manual LAS, are consistently associated with long-term mortality in patients with TS. Therefore, these findings support the use of longitudinal cardiac deformation as a marker for risk stratification, albeit clinical parameters outperformed myocardial strain in multivariable analysis. Additional data are required to determine the prognostic value regarding early and delayed cardiovascular complications and to assess management strategies based on LV longitudinal strain.

## Figures and Tables

**Table 1:** Baseline characteristics.

Variable	All Patients (*n* = 147)	Survivors (*n* = 119)	Non-Survivors (*n* = 19)	*p*
Age (years)	72 (61, 78)	71 (61, 77)	77 (72, 82)	0.006
Male sex	13/147 (8.8)	8/119 (6.7)	4/19 (21.1)	0.040
Hypertension	122/147 (83.0)	97/119 (81.5)	17/19 (89.5)	0.395
Diabetes mellitus	33/147 (22.4)	20/119 (16.8)	8/19 (42.1)	0.011
Hypercholesterolemia	31/147 (21.1)	26/119 (21.8)	5/19 (26.3)	0.665
Current smoking	28/147 (19.0)	23/119 (19.3)	3/19 (15.8)	0.714
Body mass index (kg/m^2^)	25 (22, 29)	25 (22, 29)	24 (21, 27)	0.440
Atrial fibrillation	24/147 (16.3)	14/119 (11.8)	6/19 (31.6)	0.023
Malignancy	27/147 (18.4)	17/119 (14.3)	8/19 (42.1)	0.003
Killip-class on admission				0.519
1	114/147 (7.6)	95/119 (79.8)	14/19 (73.7)	
2	25/147 (17.0)	18/119 (15.1)	5/19 (26.3)	
3	2/147 (1.4)	1/119 (0.8)	-	
4	6/147 (4.1)	5/119 (4.2)	-	
Days of hospitalization	5 (3, 7)	5 (3, 7)	4 (3, 7)	0.566
Stressful event	97/147 (66.0)	75/119 (63.0)	16/19 (84.2)	0.070
Physical trigger	60/147 (40.8)	42/119 (35.3)	14/19 (73.7)	0.002
Emotional trigger	37/147 (25.2)	33/119 (27.7)	2/19 (10.5)	0.109
ST-segment elevation	50/142 (35.2)	36/115 (31.3)	9/19 (47.4)	0.170
Ballooning pattern				0.165
Apical	96/147 (65.3)	74/119 (62.2)	16/19 (84.2)	
Midventricular	48/147 (32.7)	42/119 (35.3)	3/19 (15.8)	
Basal	3/147 (2.0)	3/119 (2.5)	-	
Initial LV-EF * (%)	47 (41, 53)	48 (41, 53)	47 (40, 49)	0.570
Follow-up LV-EF * (%)	60 (55, 66)	60 (55, 65)	58 (50, 69)	0.394
Discharge medication				
Aspirin	64/146 (43.8)	52/119 (43.7)	7/18 (38.9)	0.701
Clopidogrel	16/146 (11.0)	14/119 (11.8)	1/18 (5.6)	0.432
Oral anticoagulation	19/146 (13.0)	12/119 (10.1)	5/18 (27.8)	0.034
Beta blocker	141/146 (96.6)	115/119 (96.6)	17/18 (94.4)	0.644
ACE inhibitor/AT-R blocker	141/146 (96.6)	114/119 (95.8)	18/18 (100)	0.376
Aldosterone antagonist	32/146 (21.9)	27/119 (22.7)	3/18 (16.7)	0.565
Diuretic	80/146 (54.8)	60/119 (50.4)	14/18 (77.8)	0.030
Statin	57/146 (39.0)	46/119 (38.7)	7/18 (38.9)	0.985

Data are presented as *n*/N (%) or median (IQR). *p*-values were calculated for the comparison between survivors and non-survivors during long-term follow-up. Nine patients were lost to follow-up. * assessed with transthoracic echocardiography. ACE = angiotensin converting enzyme; AT-R = angiotensin receptor; EF = ejection fraction; LV = left ventricular.

**Table 2:** CMR findings.

Variable	All Patients (*n* = 147)	Survivors (*n* = 119)	Non-Survivors (*n* = 19)	*p*
LV ejection fraction (%)	48 (40, 53)	48 (40, 53)	47 (40, 50)	0.678
LVEDV (mL)	132 (114, 153)	131 (113, 151)	140 (121, 160)	0.200
LVESV (mL)	68 (55, 82)	68 (53, 81)	72 (60, 82)	0.403
FT-GLS (%)	−14.2 (−11.3, −18.2)	−14.5 (−11.5, −18.8)	−12.6 (−9.2, −16.4)	0.097
TT-GLS (%)	−10.4 (−8.6, −12.8)	−10.9 (−8.8, −13.3)	−8.9 (−7.6, −11.3)	0.032
LAS (%)	−11.2 (−9.1, −14.2)	−12.0 (−9.5, −14.5)	−10.2 (−7.7, −12.8)	0.014
Myocardial edema *	101/125 (80.8)	82/99 (82.8)	12/18 (66.7)	0.112
LGE	-	-	-	
LV thrombus	3/147 (2.0)	2/119 (1.7)	1/19 (5.3)	0.320
Pericardial effusion	48/147 (32.7)	37/119 (31.1)	7/19 (36.8)	0.618
Pleural effusion	63/147 (42.9)	49/119 (41.2)	11/19 (57.9)	0.172
RV involvement	41/147 (27.9)	30/119 (25.2)	8/19 (42.1)	0.126

Data are presented as *n*/N (%) or median (IQR). *p*-values were calculated for the comparison between survivors and non-survivors during long-term follow-up. Nine patients were lost to follow-up. * T2-weighted sequences with sufficient image quality available in 125/147 patients (85%). EDV = end-diastolic volume; ESV = end-systolic volume; FT = feature tracking; GLS = global longitudinal strain; LAS = long-axis strain; LGE = late gadolinium enhancement; LV = left ventricular; RV = right ventricular; TT = tissue tracking.

**Table 3:** Predictors of long-term mortality in univariate and stepwise multivariable Cox regression analysis.

Variable	Univariate		Stepwise Multivariable	
	HR (95% CI)	*p*	HR (95% CI)	*p*
Age (years)	1.07 (1.02–1.12)	0.010	-	-
Male sex	4.27 (1.36–13.48)	0.013	-	-
Diabetes mellitus	3.06 (1.22–7.68)	0.018	6.15 (2.18–17.32)	0.001
Atrial fibrillation	3.21 (1.20–8.55)	0.020	7.65 (2.21–26.51)	0.001
Malignancy	3.17 (1.27–7.92)	0.013	4.32 (1.53–12.19)	0.006
Physical trigger	3.89 (1.40–10.80)	0.009	4.77 (1.58–14.39)	0.006
Pleural effusion	2.80 (1.07–7.32)	0.035	-	-
FT-GLS (%)	1.10 (1.00–1.20)	0.041	-	-
TT-GLS (%)	1.18 (1.02–1.37)	0.026	-	-
LAS (%)	1.19 (1.03–1.36)	0.015	-	-

95% CI = confidence interval, HR = hazard ratio.

## Abbreviations

CI—Confidence interval; CoV—Coefficient of variation; CMR—Cardiac magnetic resonance; FT—Feature tracking; GLS—Global longitudinal strain; HR—Hazard ratio; ICC—Intraclass correlation coefficient; IQR—Interquartile range; LAS—Long axis strain; LV—Left ventricular; SSFP—Steady-state free precession; TS—Takotsubo syndrome; TT—Tissue tracking.
